# Recognizing the Elephant in the Room: High Arrhythmia Risk in Hemodialysis Patients' Prescriptions

**DOI:** 10.7759/cureus.111493

**Published:** 2026-06-25

**Authors:** Awanti A Meshram, Amol Bhawane, Gunjan K Ghodeshwar, Anand Chellappan

**Affiliations:** 1 Nephrology, All India Institute of Medical Sciences, Nagpur, Nagpur, IND; 2 Cardiology, All India Institute of Medical Sciences, Nagpur, Nagpur, IND

**Keywords:** arrhythmia, drug prescriptions, hemodialysis, polypharmacy, torsades de pointes

## Abstract

Background: Chronic kidney disease (CKD) patients on maintenance hemodialysis are at substantial risk of developing cardiovascular events, such as torsades de pointes and sudden cardiac death. QT-prolonging drugs have been well known, but the effect of these drugs on vulnerable populations, such as hemodialysis patients, has been understudied. This study aimed to analyse the prescription pattern for arrhythmogenic drugs in CKD patients on hemodialysis and evaluate for QT prolongation.

Methods: The cross-sectional observational study was carried out in a tertiary care teaching hospital between November 2023 and March 2024 on CKD patients on maintenance hemodialysis for over three months. The demographic profile and biochemical investigations were recorded. An echocardiogram was performed to look for structural and functional abnormalities. An electrocardiogram (ECG) was obtained, and the QTc interval was calculated using the Fridericia formula. QTc prolongation was defined as > 440 ms in males and > 460 ms in females. QT-prolonging drugs were categorized using the CredibleMeds® classification into "known," "possible," and "conditional" risk groups.

Results: Fifty CKD patients were studied, out of which polypharmacy was seen in 98% of prescriptions with a median of nine medications per prescription (interquartile range (IQR): 8-11), and 92% of patients were taking at least one QT-prolonging medication per prescription. Prolonged QT interval was seen in 42% of the patients. Diuretics and proton pump inhibitors were the most prescribed QT-prolonging medications.

Conclusion: Prescription of QT-prolonging medications is widely prevalent in hemodialysis patients, with a substantial number of them developing QT prolongation. These findings underscore the need for cautious prescribing practices in hemodialysis patients.

## Introduction

Patients with chronic kidney disease (CKD) on hemodialysis are vulnerable to complications such as sudden cardiac death (SCD), which accounts for almost 30% of all deaths. Drug-induced QT prolongation is linked to the development of fatal polymorphic ventricular tachycardia called Torsades de pointes (TdP) and SCD. Dialysis patients are susceptible due to their cardiovascular disease burden, frequent electrolyte shifts, polypharmacy, and thrice-weekly hemodialysis. Although many QT-prolonging drugs are well documented, data on their fatal effects in dialysis patients are limited. A large descriptive drug utilization study (338,515 hemodialysis patients and 40.7 million non-end-stage renal disease patients) showed that QT-prolonging drug use was 1.4-2.5 times higher in hemodialysis patients [1]. Another cohort study using United States Renal Data System (USRDS) data (2007-2017) showed azithromycin was associated with a higher risk of SCD than amoxicillin, but lower than levofloxacin [2]. Despite the known association between CKD, hemodialysis, and an increased risk of arrhythmias, there is a paucity of evidence from India regarding the prescription pattern of arrhythmogenic drugs in the hemodialysis population. The prescribing practices, disease burden, healthcare delivery systems, medication availability, and utilisation patterns differ substantially. Our study aims to analyse the prescription patterns in dialysis patients for QT-prolonging medications - a modifiable and potentially preventable risk factor for SCD.

The objectives of our study were to analyse the prescription pattern of CKD patients on hemodialysis coming to the dialysis unit and nephrology outpatient department (OPD) for drugs with arrhythmogenic potential and to perform an electrocardiogram (ECG) to assess the QT interval.

## Materials and methods

This cross-sectional study was conducted in a tertiary care teaching hospital between November 2023 and March 2024. The study was approved by the Institutional Ethics Committee (IEC/Pharmac/2023/663). Adult CKD patients on hemodialysis aged more than 18 years with a dialysis vintage of more than 90 days were included, irrespective of their comorbidities. The exclusion criteria were CKD patients not on dialysis, dialysis vintage less than 90 days, and patients admitted with intercurrent acute illness. A total of 50 patients were recruited by convenience sampling in the nephrology OPD and dialysis unit after obtaining consent. The sample size was limited to 50 participants due to the limited duration of the study, which was conducted as part of an Indian Council of Medical Research (ICMR) Short Term Studentship (STS) project aimed at promoting undergraduate research.

The following data were collected: 1) hematological (complete blood count) and biochemical parameters (blood urea, serum creatinine, serum sodium, potassium, calcium, magnesium, serum bilirubin (total and direct), aspartate transaminase (AST), alanine transaminase (ALT), serum albumin, and alkaline phosphatase (ALP)); 2) electrocardiogram obtained on a separate non-dialysis day; 3) 2D-echocardiogram; and 4) drug prescription collected from the hospital information system to evaluate for polypharmacy and QT interval prolonging medications.

The prescribed medications were classified based on the risk for prolonging the QT interval into known, possible, and conditional TdP risk based on CredibleMeds® classification [3].

All ECGs were independently evaluated by the student investigator under the supervision and guidance of the study guides, including a nephrologist and a cardiologist. The QT interval was measured from the start of the QRS complex to the end of the T wave. It was assessed in Lead II or Lead V5-6, although preference was given to the lead with the longest duration. Large U waves (> 1 mm) that are fused with the T wave were included in the measurement. Smaller T waves and those that are separated from the T wave were excluded. The maximum slope intercept method is used to define the end of the T wave. The QT interval was corrected for the heart rate using the Fridericia formula (QTc=QT/RR1/3) [4]. The criteria for QT prolongation for males and females were defined as > 440 msec and > 460 m sec, respectively [5,6].

## Results

The study included 50 hemodialysis patients, with 76% (38/50) being male (Table 1). The most common etiology was CKD of unknown origin (46%), followed by diabetes (22%).

**Table 1:** Characteristics of hemodialysis patients

Patient Characteristic	Number (%)
Gender	
Male	38 (76)
Female	12 (24)
Age Group	
18-30 years	11 (22)
31-45 years	21 (42)
46-60 years	15 (30)
61-75 years	3 (6)
Etiology of CKD	
Diabetes	11 (22)
Hypertension	4 (8)
Chronic Glomerulonephritis	7 (14)
Unknown Cause	23 (46)
Chronic Tubulointerstitial Disease	2 (4)
Congenital Anomalies of the Kidney and Urinary Tract (CAKUT)	2 (4)
Postpartum Renal Cortical Necrosis	1 (2)
Biochemical Parameters	
Parameters	Mean (Standard Deviation)
Blood urea (mg/dL)	95.54 (54.28)
Serum Creatinine (mg/dL)	8.2 (3.9)
Serum Sodium (mmol/L)	137.7 (4.7)
Serum Potassium (mmol/L)	4.77 (0.88)
Serum Calcium (mg/dL)	8.93 (1.33)
Serum Phosphorus (mg/dL)	4.74 (1.79)
Serum Magnesium (mg/dL)	2.56 (0.56)
Serum Total Protein (g/dL)	6.85 (1.07)
Serum Albumin (g/dL)	3.91 (0.75)

**Figure 1:**
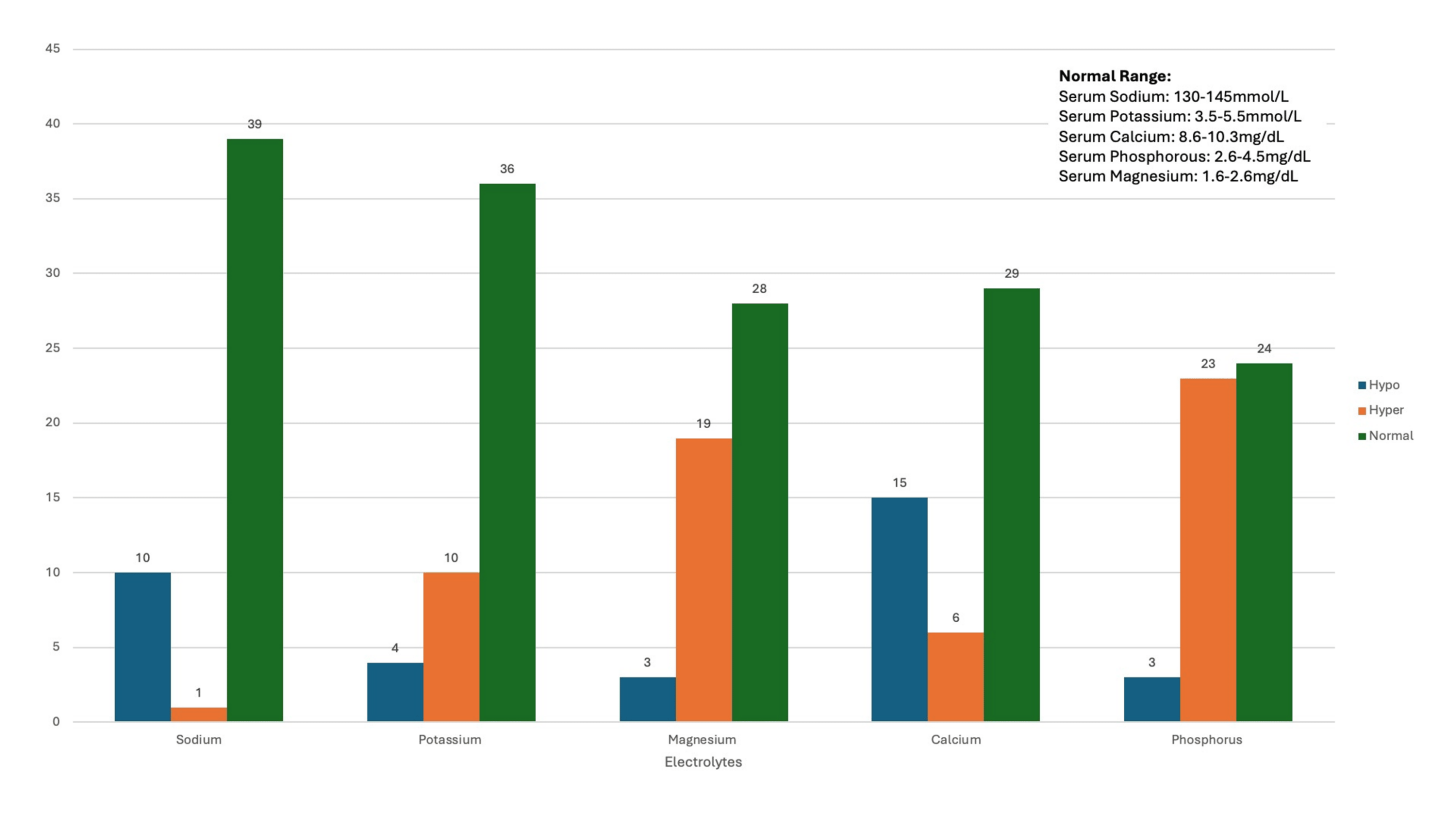
Electrolyte abnormalities in hemodialysis patients The bar chart depicts the number of participants with hypo-, hyper-, and normal levels of five electrolytes: sodium, potassium, magnesium, calcium, and phosphorus. Abbreviations: Hypo = below reference range; Hyper = above reference range. Values represent the number of participants in each category.

Anemia was highly prevalent, with the mean hemoglobin of 8.7±1.8g%, and only 6% of the participants had a hemoglobin of greater than 12 g/dL. Hyperphosphatemia (47%), hypermagnesemia (38%), and hypocalcemia (30%) were the common electrolyte abnormalities observed (Figure 1). Hyperkalemia was observed in 20% of the patients. 

Polypharmacy, defined as concomitant use of >/= 5 medications, was almost universal, seen in 98% (49/50) prescriptions [7]. The average number of drugs per prescription was 9.58±2.8 (median: 9, interquartile range (IQR): 8-11). A total of 78 different drugs were identified across prescriptions, with erythropoietin being the most prescribed. Fifteen among the 78 drugs were found to have TdP risk (Table 2).

**Table 2:** Various drugs in the prescriptions classified by their TdP risk TdP: Torsades de pointes

TdP Risk Category	Category Definition	Drugs
Known TdP Risk	Drugs that prolong the QT interval and are clearly associated with a known risk of TdP, even when taken as recommended	Escitalopram, Azithromycin, Levofloxacin, Ondansetron Hydroxychloroquine, Ivabradine
Possible TdP Risk	Drugs that can cause QT prolongation but currently lack evidence for a risk of TdP when taken as recommended	Tramadol, Alfuzosin, Levetiracetam
Conditional TdP Risk	Drugs that are associated with TdP only under certain conditions, example excessive dose in patient with conditions such as hypokalaemia or when taken with interacting drugs or drugs that create conditions that facilitate or induce TdP, example, cause electrolyte disturbance that induces TdP	Torsemide, Pantoprazole, Furosemide, Esomeprazole, Diltiazem, Metolazone

**Figure 2:**
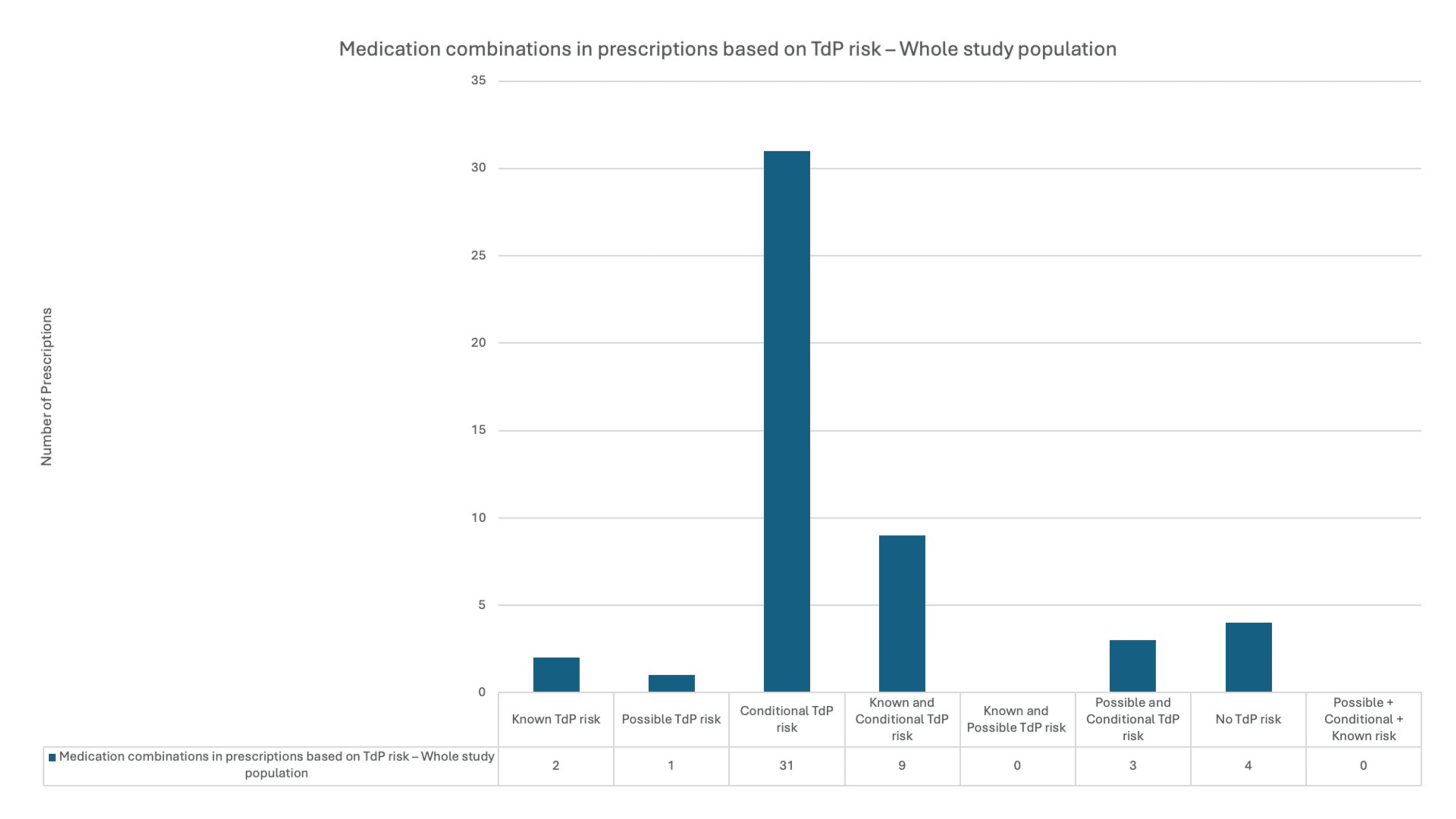
Medication combinations in prescriptions based on TdP risk - whole study population TdP: Torsades de Pointes

**Figure 3:**
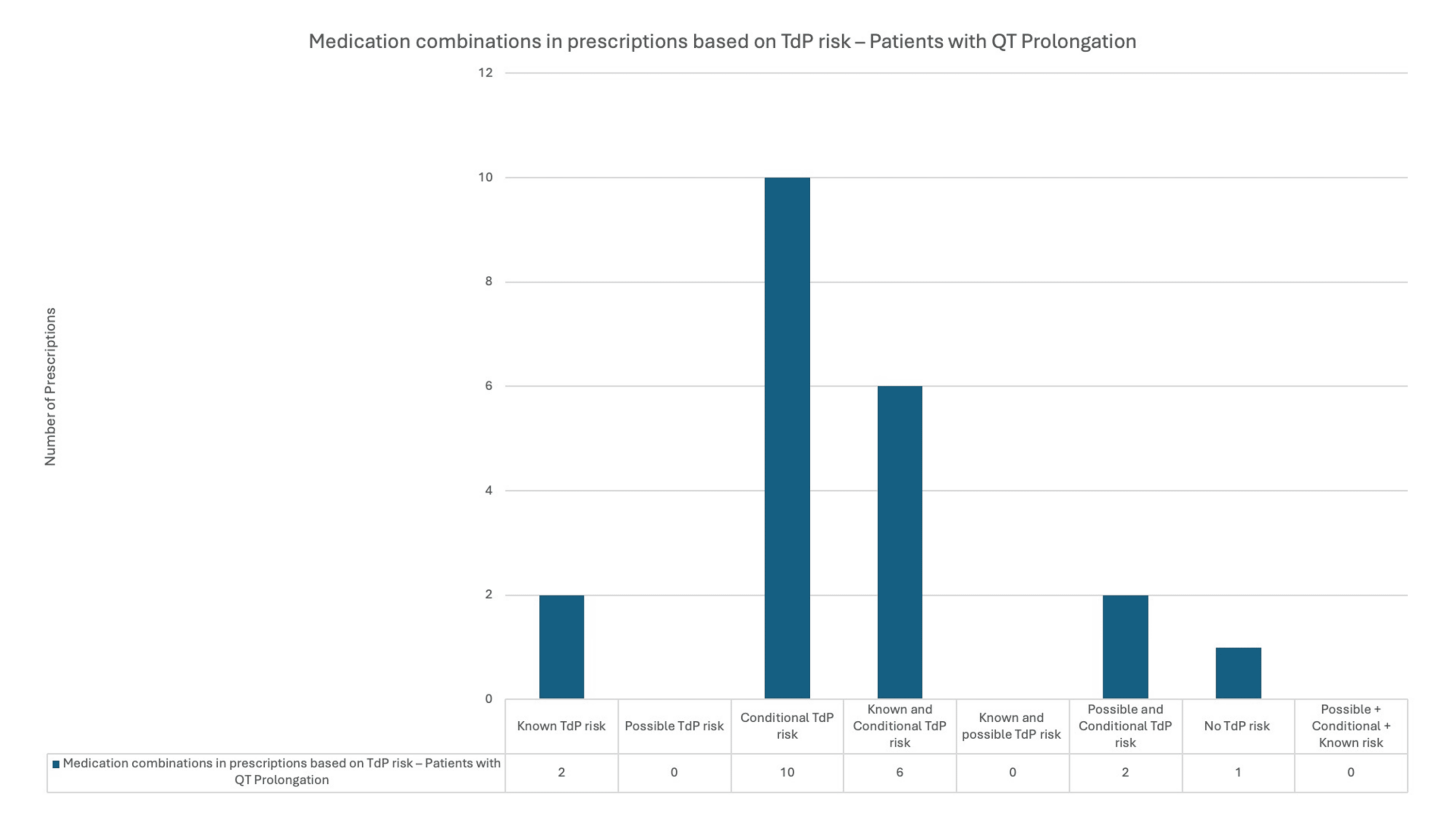
Medication combinations in prescriptions based on TdP risk - patients with QT prolongation TdP: Torsades de Pointes

The average QT-prolonging medication per prescription was 1.5 (range: 0-3), with the most common being torsemide (n-27/50), followed by pantoprazole (n-23/50). The prescription pattern of the drugs with TdP risk has been depicted in Figures 2-3. 

The mean QTc interval was 434.98 msec (±34.7), with 21 patients (male: 15, female: 6) having QTc prolongation. All except one patient were receiving QT-prolonging medications. Further, the patients with QT prolongation had an average of 1.7 QT-prolonging medications per prescription compared to 1.34 in patients with a normal QT interval.

Left ventricular hypertrophy (72%) and regional wall motion abnormalities (42%) were the echocardiographic abnormalities noted.

## Discussion

The prevalence of CKD has been steadily increasing worldwide, with a global age-standardized prevalence of 14.2% (13.4-15.2%) in adults, a relative rise of 3.5% (2.7-4.1%) from 1990 [8]. The incidence and prevalence of end-stage kidney disease patients requiring dialysis have also increased. Between 1990 and 2017, the overall age-standardized global incidence of dialysis increased by 43.1% [9]. Patients with CKD are frequently prescribed multiple medications to manage both disease-related complications and associated comorbidities. With the rising burden of CKD, these patients increasingly encounter primary care physicians for the management of concurrent medical conditions, making polypharmacy inevitable in routine clinical practice. Several commonly prescribed drugs in this population have the potential to prolong the QT interval and predispose patients to life-threatening arrhythmias, including TdP. CKD patients on dialysis may have several additional risk factors, such as electrolyte imbalances, structural heart disease, left ventricular hypertrophy, autonomic dysfunction, and reduced drug clearance. Awareness of this risk, as well as knowledge of the QT-prolonging potential of commonly used medications, is therefore essential for clinicians involved in the care of patients with CKD. Our study highlights this often overlooked yet clinically significant issue - an "elephant in the room" that may go unnoticed without deliberate consideration.

In this cross-sectional study evaluating the prescription pattern of hemodialysis patients, we found a very high prevalence of polypharmacy (98%), with 92% of the prescriptions containing at least one QT-prolonging drug. Conditional TdP risk medications were most prescribed, accounting for 86% of the prescriptions. QT was prolonged in 21 patients, and they had a 1.2 times higher prescription of drugs with TdP risk.

Our study found an average of 9.6 (±2.8) medications per prescription, such as the average of 10 (±5) reported by Kimura et al. and a median of 10 (9-13) by Chakraborty et al. [10,11]. A high polypharmacy rate of 93-97.6% has also been previously reported and is likely to be due to the burden of comorbidities in this group [12,13]. The existing literature on the prescription of QT-prolonging drugs among hemodialysis patients is sparse, especially from India. In a large study using three administrative databases (United States Renal Data System, MarketScan, and Medicare claims) consisting of 3,38,515 hemodialysis patients, patients with demographic and clinical risk factors for drug‐induced QT prolongation were exposed to medications with known TdP risk more often than patients without risk factors [1]. Annual utilization rates of QT-prolonging medications with known TdP risk in hemodialysis patients were ~1.4 to ~2.5 times higher than utilization rates in individuals without end‐stage kidney disease. However, this study did not assess the ECG, echocardiography, and serum electrolytes. Dyselectrolytemia was widely prevalent (90%) in our study. Echocardiographic abnormalities were also frequent. All these together portend a heightened risk for arrhythmias in these patients.

The strength of this study is the simultaneous evaluation of outpatient hemodialysis prescriptions for the pattern and frequency of use of drugs with TdP risk, along with ECG, echocardiography, and electrolyte analysis. The study has multiple limitations, including a cross-sectional design, a single centre with a small sample size, use of convenience sampling, lack of adjustment for confounders, and non-evaluation of dialysis-specific variables. However, the findings represent more than just the tip of an iceberg, considering the alarming prevalence of polypharmacy and frequent use of drugs with TdP risk. Future prospective studies with a larger sample size correlating with cardiovascular safety and mortality would be of utmost value in validating our findings.

## Conclusions

Our study highlights an alarming prevalence of polypharmacy (98%) and the widespread prescription of QT-prolonging medications (92%) among CKD patients on maintenance hemodialysis. With 42% of the studied cohort exhibiting QTc prolongation, the findings emphasize that these patients are at a significantly heightened risk for life-threatening arrhythmias, such as TdP, and sudden cardiac death. The risk is further compounded by the high frequency of concurrent electrolyte abnormalities and structural heart disease. Recognizing this "elephant in the room" is essential for clinicians to improve cardiovascular safety and reduce mortality in hemodialysis patients.
